# Three Basic Residues of Intracellular Loop 3 of the Beta-1 Adrenergic Receptor Are Required for Golgin-160-Dependent Trafficking

**DOI:** 10.3390/ijms15022929

**Published:** 2014-02-20

**Authors:** Catherine E. Gilbert, David M. Zuckerman, Pamela L. Currier, Carolyn E. Machamer

**Affiliations:** 1 Department of Cell Biology, Johns Hopkins University School of Medicine, 725 N. Wolfe St., Baltimore, MD 21205, USA; E-Mails: cgilbe15@jhmi.edu (C.E.G.); pamelaronco@alumni.ithaca.edu (P.L.C.); 2 Department of Molecular Microbiology and Immunology, Johns Hopkins Bloomberg School of Public Health, 615 N. Wolfe St., Baltimore, MD 21205, USA; E-Mail: dzuckerm@jhsph.edu

**Keywords:** β-1 adrenergic receptor, Golgi complex, golgin-160, trafficking

## Abstract

Golgin-160 is a member of the golgin family of proteins, which have been implicated in the maintenance of Golgi structure and in vesicle tethering. Golgin-160 is atypical; it promotes post-Golgi trafficking of specific cargo proteins, including the β-1 adrenergic receptor (β1AR), a G protein-coupled receptor. Here we show that golgin-160 binds directly to the third intracellular loop of β1AR and that this binding depends on three basic residues in this loop. Mutation of the basic residues does not affect trafficking of β1AR from the endoplasmic reticulum through the Golgi complex, but results in reduced steady-state levels at the plasma membrane. We hypothesize that golgin-160 promotes incorporation of β1AR into specific transport carriers at the *trans*-Golgi network to ensure efficient delivery to the cell surface. These results add to our understanding of the biogenesis of β1AR, and suggest a novel point of regulation for its delivery to the plasma membrane.

## 1. Introduction

G protein-coupled receptors (GPCRs) are a large family of plasma membrane localized receptors with a characteristic seven transmembrane domain topology. Through activation of heterotrimeric G-proteins, GPCRs impact a multitude of normal cellular and disease processes and thus their modulation is of great interest. The regulation of receptor desensitization, internalization, and recycling back to the membrane has been extensively studied; less understood is the regulation of receptor biosynthesis and trafficking to the plasma membrane (reviewed in [1,2]).

GPCR progression through the endoplasmic reticulum (ER) and Golgi is mediated by interactions with several classes of proteins. Several of these, including many PDZ (PSD-95, Discs-large, and ZO-1) containing proteins, interact with the *C* terminus of their GPCR binding partners, while others show specific binding to the third intracellular loops [2–5]. Recently, a highly conserved leucine in the first intracellular loop was shown to be critical for the ER export of multiple GPCRs through an unknown mechanism [6]. Receptor-activity-modifying proteins (RAMPs) not only promote ER exit of a subset of GPCRs, but can influence receptor specificity at the plasma membrane [2,7]. GPCRs also show different requirements for Ras-like small GTPases during their trafficking [8]. In this paper we focus on the atypical role of golgin-160 in influencing the initial delivery of the beta-1 adrenergic receptor (β1AR) to the plasma membrane.

β1AR is the primary β adrenergic receptor in human cardiomyocytes and is responsible for the catecholamine-induced regulation of heart rate in the sympathetic “flight or fight” response. It also has been implicated in adipogenesis and memory formation; however, the majority of research has concentrated on its role in the heart as β1AR has been widely connected with heart disease [9–11]. Both the downregulation and overstimulation of β1AR have been connected to myocyte apoptosis [12–14]. Until relatively recently, the bulk of research on the surface expression of β1AR has focused on its cycle of receptor desensitization, internalization, and recycling to the plasma membrane. This started to shift in 2004 when He *et al.* demonstrated that β1AR can be delayed at the Golgi when the Golgi resident PDZ-containing protein PIST (PDZ domain protein interacting specifically with TC10) is overexpressed [3], and in 2006 Hicks *et al.* found that golgin-160 promotes trafficking of β1AR from the Golgi complex to the plasma membrane [15].

Golgins are a large family loosely characterized by their Golgi localization and extended coiled coil motifs and have traditionally been associated with maintenance of Golgi structure and assisting bulk flow of proteins through the organelle [16]. Golgin-160 is a vertebrate-specific golgin that is one of only three golgins implicated in trafficking of specific cargoes [15,17–20]. Hicks *et al.* demonstrated *in vitro* that the third intracellular loop of β1AR can interact with the N-terminal head domain of golgin-160 in a PIST-independent manner, and that depletion of golgin-160 from HeLa cells decreases the steady state surface levels of exogenously expressed β1AR without affecting internalization rates. Surprisingly, given the steady state localization of golgin-160 to the *cis*-Golgi, this effect on trafficking occurs at or after the *trans*-Golgi network [15]. While we initially proposed that golgin-160 might promote palmitoylation of β1AR, we later demonstrated that palmitoylation of β1AR was independent of golgin-160 and instead impacts receptor internalization, leaving the mechanism by which golgin-160 facilitates trafficking of β1AR unknown [21].

The specific interaction between golgin-160 and β1AR, in addition to being an unusual role for a golgin, suggests a novel mechanism of β1AR regulation independent of receptor desensitization and recycling. In this report, we further dissect the interaction between these two proteins and demonstrate that three basic residues in the third intracellular loop of β1AR are critical for binding to golgin-160. We further show that this binding is directly responsible for the efficient delivery of exogenously expressed β1AR from the *trans*-Golgi network to the cell surface.

## 2. Results

### 2.1. Three Basic Residues in Intracellular Loop 3 of β1AR Are Required for Direct Binding to Golgin-160

**Figure 1.**
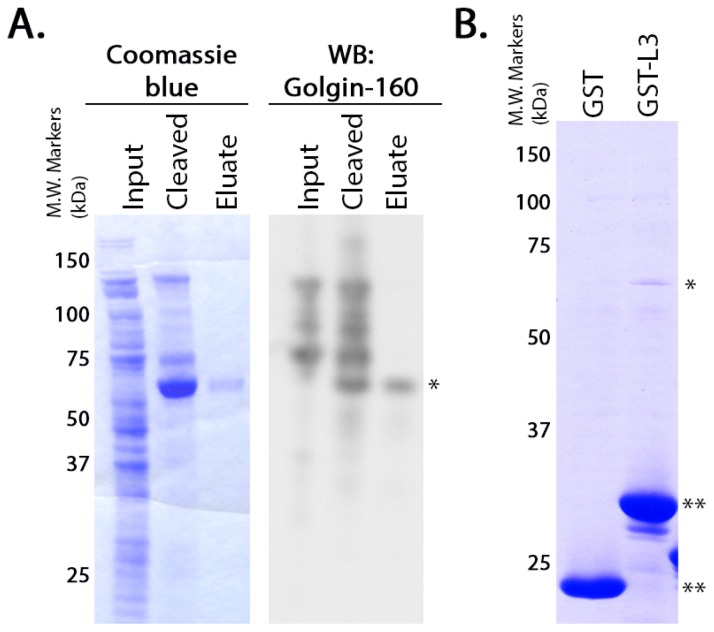
Beta-1 adrenergic receptor (β1AR) binds directly to golgin-160_(1–393)_. Representative gels for the purification of golgin-160_(1–393)_ and its binding to β1AR are shown. (**A**) The NEB IMPACT system was used to create a purified, untagged golgin-160_(1–393)_ following cleavage of the intein tag. DTT-induced cleavage caused enrichment of an approximately 60 kDa protein, which was specifically eluted off of the chitin column. This protein band could be detected using immunoblotting with an antibody to the N-terminus of golgin-160. Input, protein added to the chitin column; Cleaved, protein on the chitin column after addition of DTT but before elution; Eluate, protein released from the column after cleavage; *, golgin-160_(1–393)_; **, GST fusion proteins; (**B**) The purified, untagged golgin-160 head domain was incubated with purified GST or GST-β1AR L3 pre-bound to glutathione-Sepharose 4B beads. The beads were washed and bound golgin-160_(1–393)_ was detected by Coomassie blue staining after SDS-PAGE. Note that the samples in panel A were run on a 4%–12% gradient gel, whereas those in B were run on a 10% gel.

We previously showed that an *in vitro* transcribed and translated (IVTT) polypeptide consisting of the third cytoplasmic loop (L3, residues 249 to 325) of β1AR interacts with a region of the *N*-terminal head domain of purified GST-golgin-160, between residues 140 and 257 [15]. To demonstrate that this interaction is direct, and not the result of other proteins present in the reticulocyte lysate, we performed binding assays with purified proteins. We used the NEB Intein Mediated Purification with an Affinity Chitin-binding Tag (IMPACT) system to create a purified, untagged golgin-160 head domain (residues 1 to 393) after intein cleavage. Using this method, we purified a protein that was detectable by immunoblotting with an antibody that recognizes the *N*-terminus of golgin-160 (Figure 1A). This untagged, purified golgin-160_(1–393)_ was incubated with purified GST alone or GST-β1AR L3 that had been pre-conjugated to glutathione-Sepharose beads. Bound golgin-160_(1–393)_ was detected using Coomassie blue staining (Figure 1B). Golgin-160 bound β1AR L3 specifically, indicating a direct interaction.

**Figure 2.**
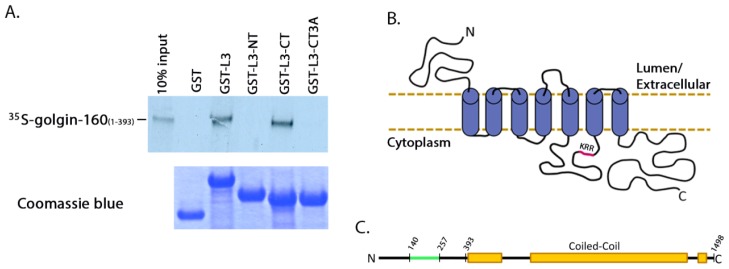
Three basic residues in the intracellular loop 3 of β1AR are required for binding to golgin-160. (**A**) Purified GST, GST-β1AR L3, GST-β1AR L3-NT, GST-β1AR L3-CT, or GST-β1AR L3-CT/3A were pre-bound to glutathione-Sepharose beads and incubated with [^35^S]-labeled golgin-160_(1–393)_. After washing the beads, bound golgin-160_(1–393)_ was detected by phosphorimaging after SDS-PAGE. The input lane represents 10% of the starting material. The GST fusion proteins were visualized for equal loading with Coomassie blue staining; (**B**) The KRR residues are located in the third intracellular loop of β1AR, shown in magenta in this flattened schematic of the receptor; (**C**) The β1AR binding region between residues 140 and 257 of golgin-160 is shown in green [15]. The coiled-coils characteristic of golgins are shown in orange.

Further characterization of the golgin-160 and β1AR binding interaction required a more robust signal, so having demonstrated that the binding is direct we returned to the IVTT-generated proteins. We obtained the strongest binding readout using small segments of GST-tagged β1AR and IVTT [^35^S]-labeled golgin-160_(1–393)_. GST was fused to the entire L3, the *N*-terminal half of L3 (residues 249 to 288, L3-NT), or the *C*-terminal half of L3 (residues 288 to 325, L3-CT). [^35^S]-labeled golgin-160_(1–393)_ was incubated with GST or the GST-L3 fusions pre-bound to glutathione-Sepharose and binding was detected by phosphorimaging. Golgin-160 binding was specific to L3-CT (Figure 2A). Human β1AR L3 contains three evolutionarily conserved basic residues, K_308_RR_310_. Through targeted mutagenesis we demonstrated that this cluster of three basic residues is required for interaction between β1AR and golgin-160: mutating the KRR sequence to three alanines (L3-CT/3A) eliminated binding *in vitro* (Figure 2A). Mutation of only two of these residues (RR_310_) to alanines did not completely eliminate binding (data not shown). A schematic of this direct binding site on β1AR is shown in Figure 2B with the critical KRR residues highlighted in magenta. Figure 2C shows a schematic of golgin-160 with its β1AR binding region highlighted in green.

### 2.2. Full Length β1AR Interacts with Golgin-160 in a KRR-Dependent Manner

**Figure 3.**
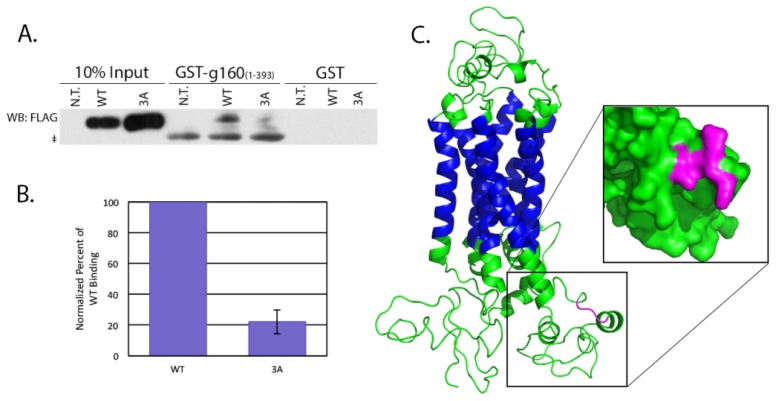
Full length β1AR interacts with golgin-160_(1–393)_ in a KRR-dependent manner. (**A**) HeLa cells were either not transfected (N.T.) or transiently transfected with constructs encoding FLAG-β1AR WT or 3A. Lysates were prepared at 18 h post-transfection and incubated with GST- or GST-golgin-160_(1–393)_-conjugated glutathione-Sepharose beads. Bound β1AR was detected by anti-FLAG immunoblotting. g160, golgin-160. The ‡ indicates nonspecific binding of the antibody to the GST-golgin-160 fusion protein; (**B**) Quantification of binding after normalization to input, *n* = 7, *p* = 1.9 × 10^−7^. Error bars represent standard deviation; (**C**) Structural model of human β1AR with an expanded region showing the surface filling model of intracellular loop 3. The KRR sequence is shown in magenta, the transmembrane regions are in blue. Structure prediction was performed using Phyre2 and modeled using PyMol.

The *in vitro* binding was performed using small fragments of β1AR attached to large protein tags, so to test the relevance of these data we performed pull downs of the full length β1AR using lysates from transiently transfected HeLa cells. GST-golgin-160_(1–393)_ was pre-conjugated to glutathione-Sepharose beads before being incubated with detergent lysates from cells transiently expressing full-length FLAG-tagged WT or 3A β1AR. β1AR bound to the golgin-160 head was detected by immunoblotting for the FLAG epitope (Figure 3A). The 3A mutant showed a 77.8% decrease in binding compared to WT (Figure 3B), supporting the *in vitro* data indicating a critical role for the KRR sequence in the interaction between these two proteins.

How does the mutation of only three residues almost completely disrupt binding? Human β1AR was modeled using Phyre2 with greater than 90% confidence for 87% of its residues using the recently crystallized turkey homologue as a template [22]. The predicted structure is displayed in Figure 3C with the KRR sequence highlighted in magenta. An expanded surface density model of intracellular loop 3 shows that the three basic residues are exposed on the surface of the loop. While the three-dimensional structure of golgin-160 is not known, the predicted globular head domain may form an acidic patch that can interact with this basic stretch, or alternatively the KRR residues may create a conformational state favorable for interaction with golgin-160.

### 2.3. Intra-Golgi Trafficking and Ligand-Induced Internalization of β1AR Are not Affected by the 3A Mutation

**Figure 4.**
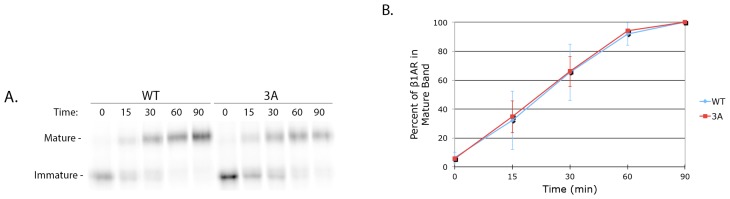
Trafficking of β1AR from the ER through the Golgi is not affected by the 3A mutation. (**A**) HeLa cells were transiently transfected with cDNAs encoding FLAG-β1AR WT or 3A. At 18 h post-transfection, cells were pulse-labeled for 15 min with [^35^S]methionine and [^35^S]cysteine, chased for 0–90 min, and lysed. β1AR was immunoprecipitated using anti-FLAG M2 affinity gel, separated by SDS-PAGE, and detected by phosphorimaging. A representative gel is shown. Passage through the *trans*-Golgi Network (TGN) was observed by a shift from an immature to a fully *O*-glycosylated (mature) band; (**B**) The percent of mature β1AR WT and 3A over 90 min. The percent mature was calculated for each individual time point by dividing the mature band density by the sum of the densities for the mature and immature bands. Error bars represent standard deviation, *n* = 3.

Previous work demonstrated that golgin-160 does not impact trafficking of newly synthesized β1AR earlier than the *trans*-Golgi Network (TGN, [15]). However, recently a triple arginine motif in the third intracellular loop of the α-2B adrenergic receptor (α_2B_AR) was found to mediate ER exit by influencing receptor interaction with the COPII-coated vesicle coat [23]. To investigate whether the 3A mutant affects the pre-TGN trafficking of β1AR, the *O*-glycosylation status of newly synthesized β1AR was examined. *O*-glycosylation is completed in the TGN, so a shift to a higher molecular weight “mature” form on denaturing gels indicates arrival of β1AR to this final Golgi compartment. A metabolic pulse-chase experiment was performed in cells expressing FLAG-β1AR WT or 3A. After a 15 min pulse label with [^35^S]methionine and [^35^S]cysteine, cells were harvested immediately (0 min) or after the indicated times of chase in unlabeled medium and β1AR was immunoprecipitated from the cell lysates. The shift from an immature to a mature, *O*-glycosylated form was visualized using phosphorimaging after SDS-PAGE and intensity of the bands measured. Three independent experiments were performed; a representative gel is shown in Figure 4A. The percent of β1AR that had reached the TGN was quantified by dividing the density of the mature band by the sum of the mature and immature band densities for each time point (Figure 4B). No difference in the rate of arrival at the TGN was observed between the WT and 3A forms of β1AR, indicating that β1AR 3A moves through the Golgi at the same rate as the wild type protein in spite of its reduced binding to golgin-160. Therefore, unlike α_2B_AR, these three basic residues do not influence ER export.

**Figure 5.**
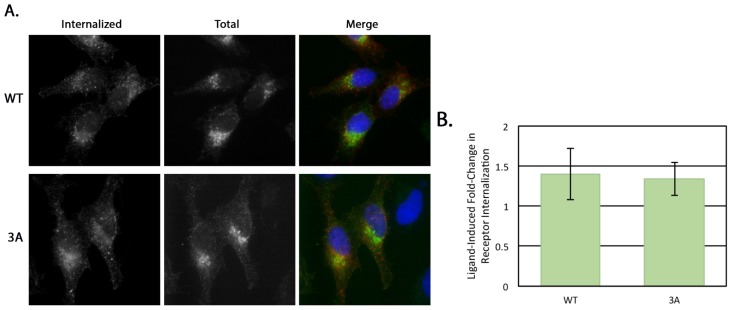
Internalization of β1AR from the plasma membrane is not affected by the 3A mutation. (**A**) Representative images of internalized β1AR WT or 3A following 30 min stimulation by Iso. HeLa cells expressing FLAG-tagged β1AR WT or 3A were serum starved for 3 h before receptor internalization was analyzed. Mouse anti-FLAG antibodies which recognize the exposed FLAG-tag were added to the serum free DMEM for 30 min, with or without the agonist Iso to induce internalization. Cells were then briefly acid washed to remove antibody from receptors still at the cell surface before they were fixed, permeabilized and labeled with a fluorescent secondary antibody to detect internalized antibody. The total internal pool of β1AR was detected using a rabbit antibody to the cytoplasmic *C*-terminus. Merge: red, anti-FLAG (internalized); green, anti-β1AR (total); blue, DNA stained with Hoechst 33258; (**B**) Internalization of β1AR WT or 3A was quantified as the fold-change of agonist-stimulated internalization over unstimulated. The fluorescent signal (integrated pixel density) was measured for each cell; only cells expressing similar levels of total β1AR were compared. Error bars represent standard deviation, *p* = 0.327.

Regulation of β1AR surface levels by endocytosis is well documented, in part due to beta adrenergic receptor agonists like isoproterenol (Iso), which stimulate receptor internalization [24]. After internalization, the receptor can either be diverted to the lysosome for degradation or recycled back to the plasma membrane. The third intracellular loop of β1AR contains a serine only two residues downstream from the KRR sequence that is critical for receptor recycling [25]; because of this, we examined whether the 3A mutation had any effect on receptor recycling using an antibody internalization assay. HeLa cells transiently expressing FLAG-tagged β1AR WT or 3A were fed anti-FLAG antibody with or without Iso for 30 min and the amount of internalized receptor was compared. Representative images are shown in Figure 5A. Internalization was quantified as the fold-increase in stimulated over unstimulated internalization in Figure 5B; no significant difference was seen between β1AR WT and 3A.

Taken together, the proper intra-Golgi trafficking of the 3A mutant (Figure 4) and normal internalization of the 3A mutant (Figure 5) indicate that disrupting the interaction between golgin-160 and β1AR does not affect the pre-TGN trafficking or plasma membrane internalization of β1AR, substantiating our golgin-160 depletion studies [15].

### 2.4. Steady State Surface Levels of β1AR 3A Are Decreased Compared to β1AR WT

**Figure 6.**
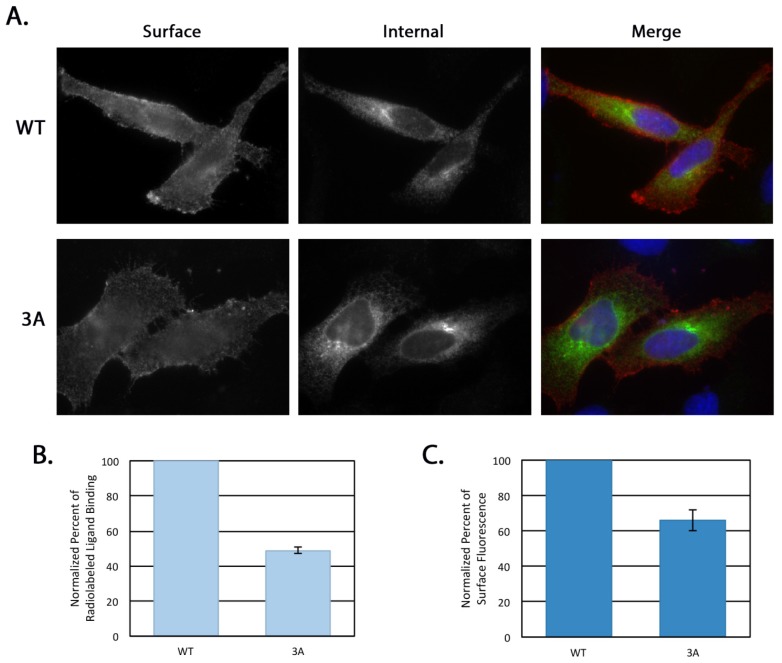
Steady state surface levels of β1AR 3A are decreased compared to β1AR WT. (**A**) Representative images of surface and internal β1AR WT or 3A at 5 h post-transfection. Cells were labeled prior to fixation and permeabilization with anti-FLAG antibody to detect surface β1AR and then after fixation and permeabilization with anti-β1AR *C*-terminus antibody, allowing for differential labeling of the surface and internal pools. Merge: red, anti-FLAG (surface); green, anti-β1AR (internal); blue, DNA stained with Hoechst 33258; (**B**) Steady state surface levels of β1AR WT and 3A in transiently transfected HeLa cells were compared using radiolabeled ligand binding at 14 h post-transfection. After incubation with ligand for 3 h at 4°C, cells were washed, lysed, and cell-associated radioactivity was determined. The surface pool of β1AR WT or 3A was calculated after subtracting background binding on non-transfected cells and normalizing for total expression level by immunoblotting. Error bars represent standard deviation, *n* = 3, *p* = 4.5 × 10^−6^; (**C**) HeLa cells were co-transfected with expression plasmids encoding cytoplasmic GFP and either FLAG-β1AR WT or 3A. Sixteen h post-transfection, FACS analysis was performed to assess surface expression of β1AR. The median fluorescence intensity for β1AR was analyzed in GFP-positive cells to represent the average amount of β1AR at the plasma membrane. Error bars represent standard deviation, *p* = 0.01.

Golgin-160 depletion by RNA interference causes a decrease in the steady state surface levels of exogenously expressed β1AR [15], so the binding-deficient 3A mutant was predicted to also have decreased surface levels. We first examined this qualitatively by differentially labeling the surface and internal pools of β1AR using indirect immunofluorescence microscopy (Figure 6A). The surface pool of the 3A mutant, labeled with anti-FLAG antibody in the cold prior to cell fixation and permeabilization, was reduced compared to WT. The internal pool, labeled using an anti-β1AR antibody post-fixation and permeabilization, appeared to be increased in the 3A mutant compared to WT. One explanation for decreased levels of protein at the plasma membrane is that β1AR 3A has a decreased half-life relative to that of the wild type protein. We ruled this out using both cycloheximide chase followed by immunoblotting and metabolic pulse-chase label followed by immunoprecipitation and phosphorimaging. The half-life of β1AR was 11.4 ± 1.6 h and the half-life of the 3A mutant was 11.0 ± 1.3 h using these two techniques. As the half-lives of both proteins were similar, we next compared the steady state surface levels of β1AR and β1AR 3A in a more quantifiable manner with a radiolabeled ligand binding assay. For this experiment, binding of [^3^H]-labeled CGP-12177, a beta adrenergic receptor agonist, was assayed in cells expressing either β1AR WT or 3A. Surface levels were determined by quantifying cell-associated radioactivity after subtraction of background binding to non-transfected cells and normalization for total expression levels by immunoblotting. The 3A mutant bound 51% less ligand than WT, implying less than half the amount of the 3A receptor was present at the cell surface compared to WT (Figure 6B).

To ensure that the reduction in signal was not due to changes in the ligand binding site, we used Fluorescence Activated Cell Sorting (FACS) analysis to measure the surface pools of β1AR. HeLa cells transiently expressing FLAG-tagged β1AR WT or 3A for 16 h were surface labeled at 4 °C using anti-FLAG antibody examined using FACS analysis. The mean fluorescence intensities of the two populations were compared as described [15]. The 3A mutant had an average of 34% less receptor at the plasma membrane compared to WT β1AR (Figure 6C). The 17% difference seen in the ligand binding assay *versus* the FACS analysis may reflect a change in the ligand binding pocket induced by the mutation, or it may be because the ligand binding assays were performed somewhat earlier post-transfection than the FACS analysis (14 h *vs.* 16 h). At 14 h, exogenously expressed β1AR may not have reached a steady state equilibrium at the plasma membrane. Nonetheless, these experiments both indicate that the 3A mutant is delivered less efficiently to the plasma membrane than the wild type protein. Further, the reductions seen in the 3A mutant surface levels are similar to the wild type β1AR surface levels after golgin-160 depletion (34% and 51% compared to 40%, [15]). This ability of the 3A mutant to phenocopy the effects of golgin-160 depletion on β1AR suggests that it is the lack of binding between golgin-160 and β1AR that causes the depletion phenotype.

## 3. Discussion

Golgin-160 was the first protein described to positively impact β1AR trafficking from the Golgi to the plasma membrane. We previously demonstrated *in vitro* binding between these two proteins [15], but the interaction was largely uncharacterized. In this report, we have shown that golgin-160 can directly bind β1AR, that three basic residues in the third intracellular loop of β1AR are critical for the interaction, and that these residues are necessary for efficient β1AR trafficking from the Golgi to the plasma membrane in cells.

The third intracellular loop and *C*-terminus are the regions of greatest variance between the β1 and β2 adrenergic receptors. It is thought that the differences in protein interactions in these two domains account for many of the functional differences between the two receptors [5]. The majority of proteins known to interact with β1AR bind to its *C*-terminal cytoplasmic tail via their PDZ domains and are involved in signal transduction and recycling from the plasma membrane [5]. Previously, only endophilins SH3p4 and SH3p13 have been shown to bind to the third intracellular loop of β1AR. This binding depends on the Src homology SH3 domain and mediates receptor internalization and coupling with G_s_ [26]. Golgin-160 binding to the third intracellular loop may influence the interactions of these other proteins with β1AR. He *et al.* found that PSD-95 can compete for a binding site with PIST via their PDZ domains in order to modulate β1AR trafficking [3]; golgin-160 could be occluding a binding site in intracellular loop 3 in a similar manner. Golgin-160 could also affect proteins binding to the *C*-terminal tail of β1AR. In 2007, Gardner *et al.* found that binding of a SAP97-AKAP79 protein scaffold to the *C*-terminus of β1AR was required for protein kinase A to phosphorylate a residue in loop 3, which is necessary for β1AR recycling to the plasma membrane [27]. In an analogous fashion, golgin-160 may work cooperatively with other proteins that interact with the *C*-terminus of β1AR. At present, the influence of golgin-160 on other β1AR binding partners has not been studied.

To date, golgin-160 is the only protein demonstrated to interact directly with β1AR to regulate its Golgi to plasma membrane trafficking. This is an atypical role for a golgin: most golgins maintain Golgi structure or assist in global trafficking, for example by acting as vesicle tethers. Only two other golgins, *trans*-Golgi-localized golgin-97 and p230/golgin-245, have been implicated in trafficking of specific cargoes. Golgin-97 colocalizes with E-cadherin in post-Golgi transport carriers and golgin-97 depletion inhibits E-cadherin trafficking [17]. Similarly, golgin-245 colocalizes with tumor necrosis factor-α (TNF) in TGN tubules and depletion of golgin-245 prevents TNF localization to the plasma membrane [20]. Golgin-160, in addition to its role in facilitating plasma membrane delivery of β1AR, can bind to the apical potassium channel ROMK and promote its delivery to the cell surface [18], and it is required for the proper localization of GLUT4 into insulin-responsive storage compartments [19].

Because golgin-160 impacts efficient trafficking of β1AR and GLUT4 is mislocalized in its absence, we hypothesize that golgin-160 facilitates specialized sorting of cargo at the TGN. The complexity of post-Golgi sorting has become more apparent in recent years. In non-polarized cells, it was once believed that proteins moved from the TGN to the plasma membrane by a single route, but it is now apparent that there are many routes to the cell surface [28]. In polarized cells, not only have distinct apical and basolateral sorting pathways been demonstrated, but proteins headed for the same cell surface also segregate into separate transport pathways at the TGN based on their mode of regulation [29–31]. Very little, however, is known about the composition of these various transport carriers or how this differential sorting occurs. We propose that golgin-160 plays a role in this sorting step, guiding cargo into vesicles so that specific cargo can be efficiently targeted. It is possible that golgin-160 accomplishes this directly by moving with cargo, or it could facilitate the interaction of its cargo with another protein, as proposed above. Both of these possibilities will be investigated by using the 3A mutant as a golgin-160-independent cargo protein. Since mutating only three basic residues dramatically interferes with this interaction, it may be that either a charged stretch or a three-dimensional conformation created by these three residues is critical for interaction with golgin-160. Our findings bring us closer to the identification of a binding motif, sequence-based or structural, among golgin-160 cargoes, which will then allow for a directed investigation into other potential cargoes including other GPCRs.

Neither depletion of golgin-160 nor disruption of its binding to β1AR completely disrupts β1AR localization—more than 50% of β1AR still makes it to the plasma membrane. This suggests that golgin-160 is playing a regulatory role, enhancing β1AR surface expression. This method of regulation is possible as golgin-160 can itself be subject to regulatory control. Golgin-160 is recruited to the Golgi through binding of GTP-bound ARF1, which is in turn rapidly cycling on and off the Golgi [32,33]. Its localization may also be affected by interactions with dynein [32]. Additionally, golgin-160 can undergo cleavage by caspase-2, -3, and -7 as well as phosphorylation by MLK3 [34,35]. All of these interactions and modifications could alter the localization or conformation of golgin-160, which could mediate interaction with β1AR. In the future, it will prove useful to study the dynamics of endogenous golgin-160 and β1AR movement in the context of cardiomyocytes to gain a better understanding of this putative regulatory cycle.

## 4. Experimental Section

### 4.1. Cells and Transfection

HeLa cells were maintained in DMEM (Life technologies, Grand Island, NY, USA) with 10% Fetal Bovine Serum (Atlanta Biologicals, Flowery Branch, GA, USA) and 0.1 mg/mL Normocin (InvivoGen, San Diego, CA, USA) at 37 °C with 5% CO_2_. Cells were grown in 35 mm dishes, 6 cm dishes, or 12-well plates depending on the experiment, and transfected with 3 μL X-tremeGENE9 DNA transfection reagent (Roche, Indianapolis, IN, USA) per 1 μg DNA.

### 4.2. Expression Constructs

Glutathione S-transferase (GST) fusion proteins of golgin-160_(1–393)_ have been previously described [36]. The untagged golgin-160_(1–393)_ protein was constructed by polymerase chain reaction (PCR) amplifying residues 1–393 from the previously described pBluescript SK+ golgin-160 construct [34] and inserting a stop codon after residue 393, which was then inserted into New England Biolab’s pTBY12 vector for purification using the IMPACT system (New England Biolabs, Beverly, MD, USA). Human FLAG-β1AR cDNA in pcDNA3 was provided by Randy Hall (Emory University, Atlanta, GA, USA). The 3A mutation, converting K_308_RR_310_ to A_308_AA_310_, was introduced using Quikchange mutagenesis (Stratagene, La Jolla, CA, USA). GST-tagged β1AR loop 3 (L3) was constructed by PCR amplification of residues 249 to 325 of β1AR and inserting the fragment into the pGEX-2T expression plasmid (GE Healthcare, Little Chalfont, Buckinghamshire, UK). GST-β1AR L3 *N*-terminus (NT) was constructed by PCR amplifying residues 249 to 288 of β1AR, and the *C*-terminus (CT) likewise by PCR amplifying residues 288 to 325, followed by insertion into the pGEX-2T expression plasmid. GST β1AR L3CT with the 3A mutation was created by amplifying residues 288 to 325 from the β1AR 3A plasmid (described above), followed by insertion into the pGET-2T expression plasmid. The cytosolic GFP vector pEGFP C1 was obtained from Clontech (Mountain View, CA, USA).

### 4.3. Antibodies

Mouse monoclonal anti-FLAG was from Sigma-Aldrich (St. Louis, MO, USA). Rabbit anti-β1AR was obtained from Santa Cruz Biotechnology (Santa Cruz, CA, USA). Rabbit anti-golgin-160 was previously described [34]. R-Phycoerythrin-conjugated IgG goat anti-mouse was from Jackson ImmunoResearch Laboratories Inc. (West Grove, PA, USA). Alexa Fluor 568 anti-mouse IgG and Alexa Fluor 488 anti-rabbit IgG were from Life Technologies (Grand Island, NY, USA). Horseradish peroxidase (HRP)-conjugated secondary antibodies were from GE Healthcare (Little Chalfont, Buckinghamshire, UK).

### 4.4. *In Vitro* Binding

GST-fusion proteins were grown in *Escherichia coli* BL21 cells (Stratagene) as previously described [36]. GST or the GST-fusion proteins were purified from the soluble fraction of the cell lysates according to manufacturer instructions using glutathione-Sepharose 4B beads (GE Healthcare, Little Chalfont, Buckinghamshire, UK). The purified proteins (5 μg each) were diluted in PBS and rebound to glutathione-Sepharose 4B beads by incubating them overnight at 4 °C with rotation. Untagged golgin-160_(1–393)_ was purified using the NEB IMPACT system as described by the manufacturer (New England Biolabs, Beverly, MD, USA). To confirm the purity and identity of the resulting protein, the eluate was separated on NuPAGE 4%–12% Bis-Tris Gel (Invitrogen, Carlsbad, CA, USA) and was detected by Coomassie blue staining or immunoblotted with rabbit anti-golgin-160. Purified golgin-160_(1–393)_ in the elution buffer (20 mM HEPES pH 8.5, 500 mM NaCl, 1 mM EDTA, 50 mM DTT) was added to the conjugated GST-fusion proteins and incubated 4–6 h at 4 °C. After washing, protein remaining attached to the glutathione-Sepharose beads was eluted, separated by SDS-PAGE (10% gel) and detected by Coomassie blue staining.

For the binding assays using *in vitro* transcribed and translated golgin-160, the GST-fusion proteins were purified and 3 μg of each was rebound to glutathione Sepharose beads as above. IVTT [^35^S]-labeled golgin-160_(1–393)_ was prepared following the manufacturer’s instructions and as previously described (Promega, Madison, WI, USA, [15]). Golgin-160 was then diluted in binding buffer (25 mM HEPES, 125 mM KOAc, 5 mM EDTA, 0.5% Nonidet P-40 (NP-40), 1 mM DTT) and incubated with the GST-fusion proteins for 2 h at 4 °C. After washing, bound golgin-160 was eluted and separated by SDS-PAGE followed by phosphorimaging on a PharosFX molecular imager (Bio-Rad, Hercules, CA, USA).

### 4.5. Pull Downs from Cell Lysates

HeLa cells grown to 70% confluency were left untransfected, or transfected with 0.5 μg of either pcDNA/WT or 3A β1AR. After incubating for an additional 16–18 h, cells were lysed in a potassium acetate lysis buffer (125 mM potassium acetate, 25 mM HEPES pH 7.1, 1 mM DTT, 1% NP-40, protease inhibitor cocktail). The soluble fraction of the lysed cells was incubated 2 h at 4 °C with 10 μg GST alone or GST-tagged golgin-160_(1–393)_ that had been pre-conjugated to glutathione-Sepharose 4B beads. After washing the beads twice with lysis buffer, protein bound to the beads was separated by SDS-PAGE and β1AR was detected by immunoblotting with anti-FLAG antibody followed by ECL. The amount of protein in the input and pull down bands was measured after imaging on a VersaDoc Imaging System Model 5000 (Bio-Rad, Hercules, CA, USA) using Quantity One volume analysis tools (Bio-Rad, Hercules, CA, USA). Percent bound was calculated after first normalizing 3A and WT to their input and significance was calculated using a heteroscedastic two-tailed Student’s *t*-test.

### 4.6. Modeling of β1AR

The nucleotide sequence of the human β1AR mRNA was obtained from NCBI for the *Homo sapiens* adrenoceptor beta 1 (ADRB1) mRNA (Accession number NM_000684). The corresponding amino acid sequence was submitted for intensive modeling to the Protein Homology/analogY Recognition Engine (PHYRE) V 2.0 server (Structural Bioinformatics Group, London, UK). The resulting predicted protein structure was then visualized using PyMOL (Schrödinger, Portland, OR, USA).

### 4.7. Metabolic Pulse-Chase and Immunoprecipitation

HeLa cells grown to 70% confluency were transiently transfected with 0.5 μg pcDNA/FLAG-β1AR WT or 3A. At 15–18 h post-transfection, cells were pulse labeled for 15 min with [^35^S]methionine and [^35^S]cysteine. For following movement of β1AR to the TGN, cells were chased for 0–90 min and lysed on ice for 5 min in 1% NP-40, 0.4% deoxycholic acid, 50 mM Tris pH 8, 62.5 mM EDTA pH 8, with protease inhibitor cocktail (Sigma-Aldrich, St. Louis, MO, USA). For determination of the half-life of β1AR, cells were chased for 0, 3, 6, or 9 h and lysed as described above. β1AR was immunoprecipiated with anti-FLAG-M2 affinity gel (Sigma-Aldrich, St. Louis, MO, USA), and after washing, the samples were eluted and separated by SDS-PAGE. The density of protein bands was measured using Quantity One volume analysis tools (Bio-Rad, Hercules, CA, USA) after phosphorimaging as described above.

### 4.8. Receptor Internalization

HeLa cells at 70% confluency on coverslips in 35 mm dishes were transfected with 0.5 μg of either pcDNA/FLAG-β1AR WT or 3A. After 16–18 h the medium was switched to serum-free DMEM for 3 h. For the internalization assay, 1 μg/mL mouse anti-FLAG antibody was added to the serum-free medium with or without 10 uM isoproterenol (Iso, Sigma-Aldrich) for 30 min at 37 °C. Cells were then washed with PBS and left untreated or remaining surface antibody was removed with an acid wash (0.5 M NaCl, 0.5% HOAc, pH 1) for 1 min at room temperature. Cells were then fixed and permeabilized as previously described [15] and the internal pool of β1AR detected using the rabbit anti-β1AR *C*-terminal antibody described above. Alexa Fluor 568 anti-mouse IgG and Alexa Fluor 488 anti-rabbit IgG were used for secondary labeling and Hoescht 33258 was used as a DNA stain. Cells with similar rabbit anti-β1AR signal intensity were selected for comparison and all images were taken on the same day with the same exposure on an Axioskop microscope (Zeiss, Thornwood, NY, USA) equipped with epifluorescence using an ORCA-03G charge-coupled device camera (Hamamatsu, Japan) using iVision software (BioVision Technologies, Exton, PA, USA). The integrated pixel density of the anti-FLAG signal for each cell was determined using Image J (National Institutes of Health, Bethesda, MD, USA) and was normalized to background fluorescence. Statistical analysis was performed using a two-tailed heteroscedastic Student’s *t*-test.

### 4.9. Surface Immunofluorescence

HeLa cells grown to 70% confluency on coverslips in 35 mm dishes were transfected with 0.5 μg of either pcDNA/FLAG-β1AR WT or 3A. After 5 h the cells were rinsed in ice cold PBS and incubated on ice with mouse anti-FLAG antibody for 15 min. Cells were then fixed and permeabilized as previously described [15] and the internal pool of β1AR detected using the rabbit anti-β1AR *C*-terminal antibody described above. Alexa Fluor 568 anti-mouse IgG and Alexa Fluor 488 anti-rabbit IgG were used for secondary labeling and Hoescht 33258 was used as a DNA stain. All images were taken on the same day with the same exposure on an Axioskop microscope (Zeiss, Thornwood, NY, USA) equipped with epifluorescence using an ORCA-03G charge-coupled device camera using iVision software (version 4.5.1, BioVision Technologies, Exton, PA, USA).

### 4.10. Cycloheximide Chase

HeLa cells were grown to 70% confluency in 35 mm dishes and were transfected with 0.5 μg of either pcDNA/FLAG-β1AR WT or 3A. After 16 h the cells were rinsed in PBS and incubated with DMEM containing 100 μg/mL cycloheximide (Calbiochem, La Jolla, CA, USA). After 0, 2, 4, 6, and 8 h, cells were lysed on ice for 5 min in 1% NP-40, 0.4% deoxycholic acid, 50 mM Tris pH 8, 62.5 mM EDTA pH 8, with protease inhibitor cocktail. Soluble protein was separated by SDS-PAGE and immunoblotted with rabbit anti-β1AR antibody.

### 4.11. Radiolabeled Ligand Binding

HeLa cells at 70% confluency on 12-well plates were transfected with 0.12 μg pcDNA/β1AR WT or 3A per well in triplicate. Nontransfected cells were used to determine background binding. After 14 h, cells were washed in cold PBS and incubated with 10 nM [^3^H]-labeled CGP-12177 (PerkinElmer Life Sciences, Waltham, MA, USA) diluted in KRH buffer (136 mM NaCl, 4.7 mM KCl, 1.25 mM MgSO_4_, 1.25 mM CaCl_2_, 20 mM HEPES, pH 7.4, and 2 mg/mL BSA) for 3 h at 4 °C. Cells were then washed in PBS and lysed as described above. Cell-associated radioactivity was determined using a Beckman Coulter LS6500 scintillation counter (Brea, CA, USA). Total expression level was determined by immunoblotting lysate prepared from a parallel transfected dish with anti-FLAG antibody followed by ECL. Three independent experiments (each done in triplicate) were averaged. Statistical analysis was performed using a two-tailed heteroscedastic Student’s *t*-test.

### 4.12. FACS Analysis

HeLa cells growing in 6 cm dishes to 70% confluency were co-transfected with 1.5 μg cytosolic GFP (pEGFP-C1) and 0.5 μg of either pcDNA/FLAG-β1AR WT or 3A. After 16–18 h, cells were harvested by trypsinization before being washed twice with DMEM and resuspended at (5–10) × 10^6^ cells/mL in blocking solution (1% BSA in PBS). After blocking for 30 min on ice the cells were incubated 1 h on ice with monoclonal mouse anti-FLAG diluted in blocking solution. Cells were washed in PBS and then were incubated 1 h on ice in phycoerythrin-labeled goat anti-mouse IgG, also diluted in blocking solution. Cells were washed again in PBS before being resuspended in serum-free DMEM and analyzed using a FACSCalibur flow cytometer (Becton Dickinson, Franklin Lake, NJ, USA). The mean phycoerythrin fluorescence intensity of cells expressing GFP was calculated for each sample using CellQuest Pro software (Becton Dickinson). Significance was determined using a two-tailed heteroscedastic Student’s *t*-test.

## 5. Conclusions

The beta-1 adrenergic receptor (β1AR) directly binds to golgin-160 and this interaction requires three basic residues, K_308_RR_310_, in the third intracellular loop of β1AR. Mutation of these residues does not alter trafficking of newly synthesized β1AR from the ER, through the Golgi or receptor internalization from the plasma membrane. However, steady state surface levels of the mutant β1AR are reduced compared to the wild type protein, indicating that binding to golgin-160 is necessary for the efficient trafficking of β1AR from the Golgi to the cell surface.
